# Time Trends in Income-related Differences in Food Group Intakes: The National Health and Nutrition Survey, Japan in 2010, 2014, and 2018

**DOI:** 10.2188/jea.JE20220220

**Published:** 2024-02-05

**Authors:** Ryoko Tajima, Mai Matsumoto, Aya Fujiwara, Xiaoyi Yuan, Chisa Shinsugi, Emiko Okada, Kayo Kurotani, Tetsuji Yokoyama, Hidemi Takimoto

**Affiliations:** 1 Department of Nutritional Epidemiology and Shokuiku, National Institutes of Biomedical Innovation, Health, and Nutrition, Tokyo, Japan; 2 Department of Epidemiology and Prevention, Center for Clinical Sciences, National Center for Global Health and Medicine, Tokyo, Japan; 3 Department of Health Sciences, Showa Women’s University, Tokyo, Japan; 4 Department of Health Promotion, National Institute of Public Health, Saitama, Japan

**Keywords:** dietary survey, nutrition policies, income related inequality, dietary records

## Abstract

**Background:**

We aimed to clarify whether differences in food group intake according to household income have changed over the last decade in Japanese people aged 20 years or older.

**Methods:**

This cross-sectional study was based on the 2010, 2014, and 2018 National Health and Nutrition Surveys in Japan. Food intake was assessed using a 1-day semi-weighed household dietary record. The participants were categorized into three groups based on their income. The mean of each food intake according to the income group was estimated by adjusting for age, occupation, and number of participants from the same household. The significance of the interaction terms between income and survey year was evaluated to assess the change in income-related differences in food intake over time.

**Results:**

Cereal intake was lower in the middle- and the highest-income groups than in the lowest-income group, regardless of sex, and the interaction between income and year was nonsignificant for cereal intake. In the former two surveys, vegetable intake was higher among the highest-income women, while in the 2018 survey, the vegetable intake decreased in the women in the middle- and the highest-income groups. The interaction between income and year was significant for vegetable intake among the women. For other foods, the differences in intake among the income groups did not significantly change over time.

**Conclusion:**

The tendency for lower cereal intake in the higher-income groups was consistent over time in both the sexes, and the tendency for higher vegetable intake in the highest income women disappeared over time.

## INTRODUCTION

Several studies have reported inequalities in the risk of non-communicable diseases and mortality according to income level.^1^^,^^2^ Diet is considered an important contributor to the income-related health inequality.^3^^,^^4^ Studies conducted in Australia^5^ and China^6^ in the 1990s, the United States^7^ and the United Kingdom^8^ in the 2000s, and Japan^9^^,^^10^ and Brazil^11^ in the 2010s, suggested that individuals with a low income consumed a low-quality diet,^7^^,^^10^ especially less consumption of fruits and vegetables.^5^^,^^6^^,^^8^^,^^9^^,^^11^ However, other studies suggested inverse association between income and diet quality. For example, during 1990s, individuals with a high income in Portugal had a lower adherence to the healthy diet based on the dietary guidelines for the prevention of chronic disease.^12^ In addition, in 2005, individuals with a high income in Mozambique had lower frequencies of vegetable consumption.^13^ These studies suggest that the association between diet quality and income might depend on the characteristics of the countries where the studies were conducted (eg, stage of economic development and degree of urbanization^14^).

However, study results may be inconsistent within the same country, since the inequalities in the dietary intakes according to income levels can be changed overtime due to several factors. For example, economic crisis as well as consequent unemployment could negatively impact dietary intake, especially in individuals with low socioeconomic status.^15^ An increase in food prices has a stronger negative impact on the demand for food in low-income households.^16^ Moreover, previous studies have suggested that public health promotion campaigns targeting the whole population might further widen socioeconomic inequality in dietary intake^17^^–^^19^; this “inequality paradox” is thought to be caused by the difference in acceptance to health promotion campaign among socioeconomic status.^20^ All of these factors can widen the income-related dietary inequalities. Considering these, understanding time trends in dietary intakes according to income levels is important, in order to update public health promotion strategies and narrow income-related dietary inequalities. For example, interventions for low-income households, such as food subsidy programs for fruits and vegetables, may be effective in some situations, but dietary problems in low-income households can change over time. Therefore, the time trends in income-related inequalities in the prevalence of unhealthy dietary habits should be monitored in each country.

Several countries have conducted repeated cross-sectional surveys to evaluate the temporal trends of income-related dietary inequalities^21^^–^^25^ and showed different findings among countries. For example, an Australian study showed that, although no dietary change was observed in vegetables across income levels and survey years, people with fruit intake ≥2 servings/day significantly decreased in the highest income level but unchanged in the lowest.^24^ On the other hand, an American study revealed that, although there was no change in inequalities in income levels over the survey years for most food groups, including vegetables, inequalities widened for the intake of whole fruit and nuts and seeds, with significantly increased intake in the highest income level and unchanged intake in the lowest.^21^ However, these time trends have not been clarified in the Japanese population, despite findings from cross-sectional studies suggesting the existence of income-related differences in food intake.^9^^,^^10^^,^^26^ In the last decade, the National Health Promotion Movement, *Health Japan 21 (second term)*, has been conducted aiming to reduce health disparities; however, the targets of healthy lifestyles including dietary habits (eg, reducing insufficient fruit and vegetable intake) were not set according to household income level. It is necessary to evaluate whether *Health Japan 21 (second term)* has been effective across all income levels. During the same period, the economic condition changed overtime in Japan. The 2008 Great Recession raised the unemployment rate (from 4% to >5%), and the Great East Japan earthquake 2011 made it stay high.^27^ Moreover, in Japan, the consumption tax increased from 5% to 8% in 2014. Therefore, these environmental changes may worsen the income-related differences in dietary habits in Japan.

The purpose of this study was to determine whether differences in food intake according to household income level increased or decreased over the last decade. This study was based on data from Japanese adults aged 20 years or older who participated in the 2010, 2014, and 2018 National Health and Nutrition Survey Japan (NHNS).

## METHODS

### Study participants

This cross-sectional study was based on the NHNS, which is conducted annually in November in the Japanese population aged ≥1 year and consisted of three parts: a dietary survey, a physical examination, and a lifestyle habits questionnaire. Detailed descriptions of survey procedures have been published previously.^28^^,^^29^ In brief, participants were recruited using a two-stage cluster sampling scheme. Households in the 300-unit blocks were randomly selected from the unit blocks of the Comprehensive Survey of Living Conditions (CSLC) for each survey year. Households with heads who were not Japanese, households that were provided with delivered/prepared meals three times a day, and one-person households residing in dormitories provided with meals were excluded from the NHNS. All participants gave verbal informed consent to the local government based on the Health Promotion Act. According to the Ethical Guidelines of Epidemiological Research, approval from the Institutional Review Board was not required.

Information on household income was collected in the 2010 NHNS for the first time, followed by in the 2011, 2014, and 2018 surveys, to obtain the baseline data of income-related differences in lifestyle factors before *the Health Japan 21 (second term)* started. Of them, this study utilized the household income data for 2010, 2014, and 2018 to equalize the time intervals. Although the individual-level participation rate was not available, the number of households participating in the dietary survey (participation rate) was 3,684 (68.8%) in 2010,^30^ 3,648 (67.2%) in 2014,^31^^,^^32^ and 3,268 (64.9%) in 2018.^33^^,^^34^ Men and women aged 20 years or older were included in this study. Among them, pregnant and lactating women, those without information regarding their household income or occupation, or those who did not participate in the dietary survey were excluded from the analyses.

### Food group intakes

A 1-day semi-weighed household dietary record was collected on a usual day, excluding Sundays and public holidays.^29^ Trained fieldworkers visited each household, provided a recording form, and instructed the main record keepers in the household (those who mainly prepared meals) about the survey purpose and how to complete the dietary record. The main record keepers were asked to weigh the foods and beverages and record their names and amounts, including the amount of food wasted and leftovers. When household members shared foods from the same dish, the approximate proportions of foods consumed by each member were also recorded. If weighing was difficult (eg, restaurant meals or school lunch), the main record-keepers were asked to record as much information as possible, including meal ingredients and estimated portion sizes. Information on sex, date of birth, pregnancy or lactation status, and occupation were also collected using the same recording form. Fieldworkers visited each household again, checked the completeness of the recording form, and confirmed missing information, if necessary. Detailed information of the records and data collection have been described in a previous study.^29^

The following food groups were used in this study: cereals, vegetables, fruits, fish and shellfish, meat, eggs, soy and other beans, milk and dairy products, snacks and confectionaries, alcoholic beverages, and tea, coffee, and other soft drinks. Foods were classified according to the food grouping methods used in the Standard Tables of Food Composition (STFC) in Japan (5^th^ revised and enlarged edition for 2010 survey,^35^ 2010 edition for 2014 survey,^36^ and 2015 edition including supplementary editions 2016 and 2017 for 2018 survey^37^). Consumption of vegetables was calculated by summing the intake of green and yellow vegetables, other vegetables, pickled vegetables, mushrooms, and seaweeds. Juice and jam were excluded from the calculation of fruit and vegetable intake, although the inclusion of these items did not change the study results (data not shown). Energy intake was calculated using the STFC in Japan. Energy-adjusted food intake (g/1,000 kcal) was calculated using the density method.^38^

The validity of dietary intake from this household-based dietary record was investigated in 64 Japanese volunteers (female students taking a dietetics course and their mothers).^39^ Dietary intakes among students estimated from the 1-day household-based dietary records kept by their mothers were compared to those estimated from the 1-day individually-based dietary records, which were independently conducted by the students. The study suggested that the household-based dietary record underestimated energy intake by 6.2% compared to individually-based dietary records, and the Pearson’s correlation coefficient between the two methods was 0.90 for energy. However, the validity of the estimated food intake was not reported in this study.

Occupation was categorized into four categories considering the average income level^40^ and the types of work (office work or hard labor); “professional/manager”, “sales/service/clerical”, “security/transportation/labor”, “and non-worker”, as well as previous studies.^10^^,^^41^

### Income and number of household members

In the 2010 and 2014 surveys, the four options provided with regards to annual household income were: “<2 million yen,” “2 to <6 million yen,” “≥6 million yen,” and “I don’t know”.^30^^,^^31^ In the 2018 survey, the five options provided were: “<2 million yen,” “2 to <4 million yen,” “4 to <6 million yen,” “≥6 million yen” and “I don’t know”.^33^ Household members who answered “I don’t know” were excluded from the analyses.

Information on the number of household members was not collected in the 2010 and 2014 surveys. In the 2018 survey, the person representing the household indicated the number of household members.^33^ Thus, the number of survey participants from the same household was used as an alternative indicator for the number of household members. In the 2018 survey, the number of household members reported by household representatives was same as survey participants from the same household in 88.5% of the participating households, and one more household member was reported in 7.3% of the participating household.

Only the household representatives were asked to provide information on household income, and the response was allocated to the other members. When several members from the same household provided information on household income, all their responses were considered invalid (ie, missing).^10^ This is because it was not possible to determine which household members had correctly answered the question.

### Statistical analyses

The analyses were performed separately for men and women. Participants were categorized into three groups according to their income: lowest (<2 million yen), middle (2 to <6 million yen), and highest (≥6 million yen). All analyses were conducted using the SAS statistical software (version 9.4; SAS Institute Inc. Cary, NC, USA). *P* values <0.05 based on the two-sided test statistics were considered statistically significant.

### Change in income-related differences in food group intakes over time

Considering that age group, occupation, and cohabitation with other family members might be associated with both dietary habits^10^^,^^42^^,^^43^ and dietary cost,^41^ all analyses were adjusted for age (ie, 20–29, 30–39, 40–49, 50–59, 60–69, and ≥70 years), occupation (ie, professional/manager, sales/service/clerical, security/transportation/labor, and non-worker), and the number of participants from the same household (ie, 1, 2, ≥3). For each survey year, the adjusted mean of each food group intake according to income was estimated using the PROC SURVEYREG analysis with STRATA (for prefecture) and CLUSTER (for unit blocks) statements to properly account for the two-stage cluster sampling scheme used in the NHNS. Tests for differences in food groups between income groups were performed by including the categories of income as dummy variables, using the lowest income category as a reference in the regression models. Differences in food group intake among the survey years were evaluated by adding survey year as dummy variables using 2010 as a reference in the model. The significance of the interaction terms between income and survey year was evaluated to assess the change in income-related differences in food intake over time.^21^^,^^24^^,^^25^ Interaction terms between income (dummy variables) and survey year (dummy variables) were added into the models, including income, survey year, age, occupation, and the number of participants from the same household. The overall significance (*P* < 0.05) of the interaction terms was evaluated using the Wald test.

Considering previous studies suggesting that household members of the same gender share dietary habits,^44^ multiple participation from the same household could result in underestimation of the standard error of food intakes according to household income levels. Therefore, we also conducted the same analyses limiting participants to a single man and single woman from the same household.

## RESULTS

**Figure 1.**
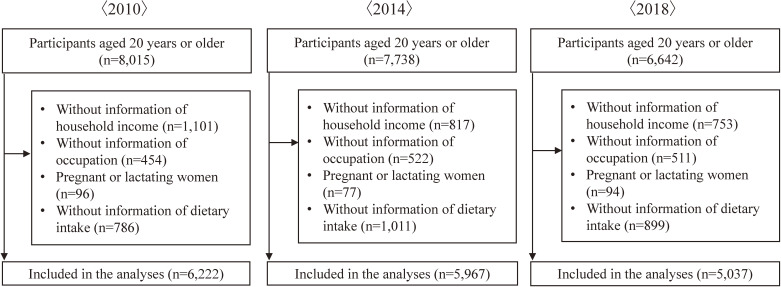
Number of participants included in the present analysis. Part of the excluded participants had missing values for ≥2 variables.

In 2010, there were 8,015 participants aged 20 or older, 7,738 in 2014, and 6,642 in 2018 (Figure 1). After excluding participants who did not meet the inclusion criteria, 6,222 adults in 2010, 5,967 adults in 2014, and 5,037 adults in 2018 were included in the analyses. Regardless of sex and survey year, the prevalence of participants in each age category, occupation, and the number of participants from the same household were significantly different among the income groups (Table 1 and Table 2). The proportion of people over 70 years old, non-workers, and single participant from the same household was the highest in the lowest-income group. Except for men in 2010 and women in 2014, the prevalence of harmful drinking differed between the income groups. Comparing those included in the analyses with those excluded from the analyses, there were differences in the prevalence of participants in each age category, income (in 2010 and 2018), occupation (in 2010 and 2018), current smoking status (in 2010 and 2014), and median intake of cereals, vegetables (in 2010 and 2014), fruits, and milk and dairy products (eTable 1).

**Table 1.** Characteristics of Japanese men according to income^a^: the 2010, 2014, and 2018 National Health and Nutrition Survey (NHNS), Japan

	2010 (*n* = 2,922)							2014 (*n* = 2,801)							2018 (*n* = 2,368)						

	Lowest income		Middle income		Highest income		*P* ^b^	Lowest income		Middle income		Highest income		*P* ^b^	Lowest income		Middle income		Highest income		*P* ^b^
	*n*	%	*n*	%	*n*	%		*n*	%	*n*	%	*n*	%		*n*	%	*n*	%	*n*	%	
	453	100.0	1,715	100.0	754	100.0		423	100.0	1,620	100.0	758	100.0		281	100	1,259	100	828	100	
Age							<0.01							<0.01							<0.01
20–29 years	27	6.0	162	9.4	74	9.8		20	4.7	93	5.7	76	10.0		5	1.8	99	7.9	84	10.1	
30–39 years	45	9.9	303	17.7	107	14.2		29	6.9	210	13.0	100	13.2		16	5.7	135	10.7	118	14.3	
40–49 years	33	7.3	243	14.2	176	23.3		30	7.1	224	13.8	159	21.0		25	8.9	180	14.3	199	24.0	
50–59 years	52	11.5	216	12.6	205	27.2		39	9.2	187	11.5	193	25.5		21	7.5	146	11.6	208	25.1	
60–69 years	132	29.1	399	23.3	113	15.0		117	27.7	443	27.3	127	16.8		67	23.8	299	23.7	127	15.3	
≥70 years	164	36.2	392	22.9	79	10.5		188	44.4	463	28.6	103	13.6		147	52.3	400	31.8	92	11.1	
Occupation							<0.01							<0.01							<0.01
Professional/manager	21	4.6	294	17.1	316	41.9		29	6.9	217	13.4	307	40.5		11	3.9	200	15.9	340	41.1	
Sales/service/clerical	59	13.0	374	21.8	198	26.3		44	10.4	321	19.8	170	22.4		39	13.9	303	24.1	178	21.5	
Security/transportation/labor	151	33.3	572	33.4	142	18.8		97	22.9	535	33.0	177	23.4		56	19.9	354	28.1	210	25.4	
Non-worker	222	49.0	475	27.7	98	13.0		253	59.8	547	33.8	104	13.7		175	62.3	402	31.9	100	12.1	
Number of participants from the same household							<0.01							<0.01							<0.01
1	86	19.0	120	7.0	23	3.1		117	27.7	125	7.7	19	2.5		109	38.8	136	10.8	32	3.9	
2	205	45.3	576	33.6	153	20.3		193	45.6	575	35.5	154	20.3		112	39.9	518	41.1	137	16.5	
≥3	162	35.8	1,019	59.4	578	76.7		113	26.7	920	56.8	585	77.2		60	21.4	605	48.1	659	79.6	

Current smoking^c^	440	100.0	1,679	100.0	739	100.0	0.22	411	100.0	1,604	100.0	755	100.0	0.52	277	100.0	1,249	100.0	819	100.0	0.43
No	299	68.0	1,103	65.7	511	69.1		290	70.6	1,112	69.3	510	67.5		190	68.6	905	72.5	587	71.7	
Yes	141	32.0	576	34.3	228	30.9		121	29.4	492	30.7	245	32.5		87	31.4	344	27.5	232	28.3	
Harmful drinking habits^d^	445	100.0	1,700	100.0	748	100.0	0.061	415	100.0	1,603	100.0	755	100.0	<0.01	278	100.0	1,249	100.0	819	100.0	<0.01
No	388	87.2	1,436	84.5	614	82.1		370	89.2	1,333	83.2	630	83.4		248	89.2	1,080	86.5	661	80.7	
Yes	57	12.8	264	15.5	134	17.9		45	10.8	270	16.8	125	16.6		30	10.8	169	13.5	158	19.3	

^a^Participants were categorized into three groups according to income: lowest (<2 million yen), middle (2 to <6 million yen), and highest (≥6 million yen).

^b^Pearson’s chi-squared test.

^c^Participants who responded as “smoking every day” or “occasionally smoking”.

^d^Defined according to the definition used in the NHNS report (33): participants who responded to the question on drinking frequencies and quantities as “every day and ≥2 units/day”, “5–6 days/week and ≥2 units/day”, “3–4 days/week and ≥3 units/day”, “1–2 days/week and ≥5 units/day”, or “1–3 days/month and ≥5 units/day”.

**Table 2.** Characteristics of Japanese women according to income^a^: the 2010, 2014, and 2018 National Health and Nutrition Survey (NHNS), Japan

	2010 (*n* = 3,300)							2014 (*n* = 3,166)							2018 (*n* = 2,669)						

	Lowest income		Middle income		Highest income		*P* ^b^	Lowest income		Middle income		Highest income		*P* ^b^	Lowest income		Middle income		Highest income		*P* ^b^
	*n*	%	*n*	%	*n*	%		*n*	%	*n*	%	*n*	%		*n*	%	*n*	%	*n*	%	
	669	100.0	1,824	100.0	807	100.0		617	100.0	1,735	100.0	814	100.0		451	100.0	1,340	100.0	878	100.0	
Age							<0.01							<0.01							<0.01
20–29 years	35	5.2	136	7.5	105	13.0		18	2.9	105	6.1	84	10.3		9	2.0	76	5.7	94	10.7	
30–39 years	44	6.6	307	16.8	116	14.4		27	4.4	220	12.7	90	11.1		11	2.4	135	10.1	107	12.2	
40–49 years	44	6.6	242	13.3	193	23.9		49	7.9	254	14.6	187	23.0		34	7.5	178	13.3	215	24.5	
50–59 years	74	11.1	279	15.3	194	24		61	9.9	244	14.1	192	23.6		41	9.1	187	14.0	222	25.3	
60–69 years	178	26.6	448	24.6	93	11.5		163	26.4	463	26.7	118	14.5		99	22.0	346	25.8	109	12.4	
≥70 years	294	43.9	412	22.6	106	13.1		299	48.5	449	25.9	143	17.6		257	57.0	418	31.2	131	14.9	
Occupation							<0.01							<0.01							<0.01
Professional/manager	34	5.1	176	9.6	122	15.1		24	3.9	132	7.6	148	18.2		16	3.5	160	11.9	174	19.8	
Sales/service/clerical	156	23.3	516	28.3	294	36.4		135	21.9	525	30.3	248	30.5		102	22.6	367	27.4	334	38.0	
Security/transportation/labor	60	9.0	135	7.4	39	4.8		38	6.2	147	8.5	71	8.7		35	7.8	113	8.4	55	6.3	
Non-worker	419	62.6	997	54.7	352	43.6		420	68.1	931	53.7	347	42.6		298	66.1	700	52.2	315	35.9	
Number of household members							<0.01							<0.01							<0.01
1	224	33.5	132	7.2	14	1.7		250	40.5	130	7.5	16	2.0		217	48.1	119	8.9	17	1.9	
2	241	36.0	634	34.8	156	19.3		250	40.5	622	35.9	160	19.7		153	33.9	581	43.4	152	17.3	
≥3	204	30.5	1,058	58.0	637	78.9		117	19.0	983	56.7	638	78.4		81	18.0	640	47.8	709	80.8	
Current smoking^c^	658	100.0	1,796	100.0	798	100.0	0.31	612	100.0	1,724	100.0	808	100.0	0.061	448	100.0	1,330	100.0	871	100.0	0.10
No	601	91.3	1,628	90.6	738	92.5		556	90.8	1,567	90.9	756	93.6		404	90.2	1,240	93.2	802	92.1	
Yes	57	8.7	168	9.4	60	7.5		56	9.2	157	9.1	52	6.4		44	9.8	90	6.8	69	7.9	
Harmful drinking habits^d^	661	100.0	1,809	100.0	799	100.0	0.026	614	100.0	1,723	100.0	809	100.0	0.052	448	100.0	1,330	100.0	871	100.0	<0.01
No	621	93.9	1,682	93.0	723	90.5		571	93.0	1,565	90.8	722	89.2		417	93.1	1,223	92.0	770	88.4	
Yes	40	6.1	127	7.0	76	9.5		43	7.0	158	9.2	87	10.8		31	6.9	107	8.0	101	11.6	

^a^Participants were categorized into three groups according to income: lowest (<2 million yen), middle (2 to <6 million yen), and highest (≥6 million yen).

^b^Pearson’s chi-squared test.

^c^Participants who responded as “smoking every day” or “occasionally smoking”.

^d^Defined according to the definition used in the NHNS report (33): participants who responded to questions on drinking frequencies and quantities as “every day and ≥1 unit/day”, “5–6 days/week and ≥1 unit/day”, “3–4 days/week and ≥1 units/day”, “1–2 days/week and ≥3 units/day”, or “1–3 days/month and ≥5 units/day”.

### Change in income-related inequalities in food intakes over time

The adjusted mean intakes of food groups according to income and survey year are presented in Table 3 and Table 4. Regardless of sex, the cereal intake was lower in the middle- and the highest-income groups than in the lowest-income group. Compared with 2010, regardless of sex and income, a lower cereal intake was observed in 2018. For both sexes, the interaction between income and survey year was not significant for cereal intake. In the 2010 and 2014 surveys, vegetable intake was higher in women with the highest income than in women with the lowest income. In women with middle- and the highest-income, vegetable intake was lower in 2018 than in 2010. The interaction between income and survey year was significant for vegetable intake among women (*P* = 0.04). In the 2010 survey, egg intake was lower in the middle- and the highest-income men than in the lowest-income men. In the 2018 survey, egg intake was higher in the middle-income men than that in the lowest-income men. In men with middle and high incomes, egg intake was higher in 2018 than in 2010. The interaction between income and survey year was significant for egg intake in men (*P* = 0.002). Except for women in the 2010 survey, the consumption of milk and dairy products was higher in the highest-income group than in the lowest-income group. In men in the lowest- and middle-income groups, milk and dairy product intake was higher in 2018 than in 2010. The interaction between income and survey year was not significant for milk and dairy products.

**Table 3.** Adjusted mean intakes^a^ of food groups according to income^b^ and survey year (men)

	2010 (*n* = 2,922)			2014 (*n* = 2,801)			2018 (*n* = 2,368)			*P* for 2014^c^	*P* for 2018^c^	*P*-interaction^d^

	*n*	Adjusted mean	SE	*n*	Adjusted mean	SE	*n*	Adjusted mean	SE			
Cereal (g/1,000 kcal)												
Lowest income	453	269.0	4.4	423	265.7	5.1	281	245.6	5.7	0.63	<0.001	
Middle income	1,715	250.8	2.4	1,620	243.5	3.0	1,259	230.5	2.9	0.20	<0.001	0.71
Highest income	754	242.7	3.7	758	233.9	4.2	828	226.3	3.4	0.27	<0.001	
*P* for middle income^e^		<0.001			<0.001			0.021				
*P* for highest income^e^		<0.001			<0.001			0.008				
Vegetable (g/1,000 kcal)												
Lowest income	453	135.4	4.8	423	128.9	5.2	281	129.3	5.7	0.27	0.12	
Middle income	1,715	134.7	2.7	1,620	139.0	3.1	1,259	133.3	2.5	0.93	0.017	0.55
Highest income	754	144.9	3.8	758	152.0	4.3	828	135.7	3.5	0.49	0.013	
*P* for middle income^e^		0.89			0.078			0.49				
*P* for highest income^e^		0.12			<0.001			0.35				
Fruit (g/1,000 kcal)												
Lowest income	453	33.0	2.6	423	30.0	3.1	281	27.4	3.9	0.72	0.59	
Middle income	1,715	38.0	1.6	1,620	37.4	2.0	1,259	32.4	1.7	0.91	0.037	0.52
Highest income	754	41.5	2.3	758	37.6	2.4	828	31.5	1.9	0.17	<0.001	
*P* for middle income^e^		0.051			0.048			0.25				
*P* for highest income^e^		0.011			0.057			0.39				
Fish and shellfish (g/1,000 kcal)												
Lowest income	453	40.0	2.0	423	37.6	2.2	281	35.2	2.6	0.30	0.15	
Middle income	1,715	39.3	1.2	1,620	38.5	1.3	1,259	32.3	1.2	0.44	<0.001	0.24
Highest income	754	41.3	1.8	758	35.2	1.8	828	32.1	1.6	0.003	<0.001	
*P* for middle income^e^		0.74			0.69			0.32				
*P* for highest income^e^		0.64			0.40			0.33				
Meat (g/1,000 kcal)												
Lowest income	453	41.0	1.6	423	49.9	2.1	281	55.3	2.8	0.003	<0.001	
Middle income	1,715	45.4	1.1	1,620	50.9	1.3	1,259	59.2	1.5	0.004	<0.001	0.20
Highest income	754	46.3	1.6	758	55.8	1.6	828	60.4	1.7	<0.001	<0.001	
*P* for middle income^e^		0.011			0.66			0.22				
*P* for highest income^e^		0.020			0.031			0.14				
Egg (g/1,000 kcal)												
Lowest income	453	20.6	1.0	423	15.7	1.0	281	17.3	1.4	<0.001	0.28	
Middle income	1,715	17.1	0.5	1,620	17.4	0.6	1,259	20.9	0.8	0.35	<0.001	0.0020
Highest income	754	17.5	0.8	758	17.7	0.8	828	20.8	1.0	0.87	0.008	
*P* for middle income^e^		<0.001			0.11			0.027				
*P* for highest income^e^		0.009			0.14			0.061				
Soy and other beans (g/1,000 kcal)												
Lowest income	453	30.2	2.4	423	32.0	2.8	281	28.4	3.3	0.50	0.89	
Middle income	1,715	28.6	1.3	1,620	29.6	1.4	1,259	29.8	1.5	0.81	0.62	0.93
Highest income	754	28.2	1.8	758	31.6	1.9	828	32.3	2.1	0.40	0.28	
*P* for middle income^e^		0.45			0.43			0.70				
*P* for highest income^e^		0.49			0.91			0.33				
Milk and dairy products (g/1,000 kcal)												
Lowest income	453	34.2	3.1	423	39.1	3.8	281	41.6	4.5	0.22	0.031	
Middle income	1,715	43.4	2.2	1,620	47.0	2.3	1,259	48.8	2.3	0.14	0.015	0.84
Highest income	754	46.9	3.5	758	49.4	3.0	828	53.1	3.1	0.37	0.29	
*P* for middle income^e^		0.003			0.040			0.15				
*P* for highest income^e^		0.002			0.025			0.030				
Snacks and confectionaries (g/1,000 kcal)												
Lowest income	453	10.1	1.1	423	10.2	1.2	281	9.2	1.2	0.88	0.78	
Middle income	1,715	8.8	0.6	1,620	10.1	0.8	1,259	9.9	0.7	0.04	0.14	0.49
Highest income	754	9.2	0.9	758	9.2	0.9	828	11.1	0.9	0.78	0.24	
*P* for middle income^e^		0.27			0.90			0.63				
*P* for highest income^e^		0.50			0.46			0.21				
Alcoholic beverages (g/1,000 kcal)												
Lowest income	453	73.9	7.4	423	71.7	8.2	281	95.1	9.1	0.91	0.14	
Middle income	1,715	89.0	5.0	1,620	101.8	5.9	1,259	85.5	5.1	0.04	0.65	0.12
Highest income	754	100.1	7.4	758	108.0	7.3	828	95.0	7.7	0.44	0.91	
*P* for middle income^e^		0.044			<0.001			0.33				
*P* for highest income^e^		0.0045			0.0012			0.99				
Tea, coffee, and other soft drinks (g/1,000 kcal)												
Lowest income	453	260.7	12.4	423	252.5	14.9	281	284.5	15.7	0.35	0.44	
Middle income	1,715	276.4	7.6	1,620	278.6	8.7	1,259	265.6	8.0	0.30	0.46	0.68
Highest income	754	282.2	11.4	758	290.8	15.0	828	275.3	10.1	0.70	0.66	
*P* for middle income^e^		0.20			0.056			0.26				
*P* for highest income^e^		0.17			0.036			0.63				

SE, standard error.

^a^Estimated by SAS PROC SURVEYREG with STRATA (for prefecture) and CLUSTER (for unit blocks) statements. The following variables were adjusted: age (20–29, 30–39, 40–49, 50–59, 60–69, and ≥70 years), occupation (professional/manager, sales/service/clerical, security/transportation/labor, and non-worker), and number of participants from the same household (1, 2, or ≥3).

^b^Participants were categorized into three groups according to income: lowest (<2 million yen), middle (2 to <6 million yen), and highest (≥6 million yen).

^c^Survey year as dummy variables using 2010 as a reference were included in the model with adjustment for above mentioned variables.

^d^*P*-values for *the income (dummy variables)* × *survey year (dummy variables)* interaction terms were calculated with adjustment for the above-mentioned variables; a significant interaction meant that the *income-related differences* in food intake changed over time.

^e^Income as dummy variables using the lowest group as a reference were included in the model with adjustment for above mentioned variables.

**Table 4.** Adjusted mean intakes^a^ of food groups according to income^b^ and survey year (women)

	2010 (*n* = 3,300)			2014 (*n* = 3,166)			2018 (*n* = 2,669)			*P* for 2014^c^	*P* for 2018^c^	*P* interaction^d^

	*n*	Adjusted mean	SE	*n*	Adjusted mean	SE	*n*	Adjusted mean	SE			
Cereal (g/1,000 kcal)												
Lowest income	669	237.0	3.4	617	235.3	3.8	451	219.9	4.7	0.44	<0.001	
Middle income	1,824	226.6	2.6	1,735	219.1	2.6	1,340	208.1	3.2	0.069	<0.001	0.30
Highest income	807	217.4	3.6	814	208.3	3.5	878	200.2	4.0	0.082	<0.001	
*P* for middle income^e^		0.010			<0.001			0.013				
*P* for highest income^e^		<0.001			<0.001			0.002				
Vegetable (g/1,000 kcal)												
Lowest income	669	176.5	5.3	617	180.3	5.0	451	173.9	6.3	0.99	0.24	
Middle income	1,824	174.9	3.5	1,735	180.0	3.3	1,340	164.6	4.0	0.052	0.042	0.04
Highest income	807	191.3	5.1	814	195.7	5.4	878	162.0	5.1	0.11	<0.001	
*P* for middle income^e^		0.79			0.95			0.17				
*P* for highest income^e^		0.042			0.029			0.16				
Fruit (g/1,000 kcal)												
Lowest income	669	57.4	3.3	617	53.6	3.4	451	47.9	3.5	0.98	0.20	
Middle income	1,824	61.3	2.3	1,735	60.0	2.2	1,340	52.9	2.3	0.73	0.009	0.995
Highest income	807	63.2	3.3	814	63.4	3.3	878	55.5	2.6	0.49	0.095	
*P* for middle income^e^		0.29			0.11			0.22				
*P* for highest income^e^		0.17			0.045			0.077				
Fish and shellfish (g/1,000 kcal)												
Lowest income	669	41.5	2.1	617	37.4	2.2	451	35.1	2.7	0.27	0.20	
Middle income	1,824	41.4	1.3	1,735	40.3	1.4	1,340	34.8	1.4	0.83	0.010	0.12
Highest income	807	44.9	2.1	814	36.7	1.8	878	34.7	1.9	0.001	<0.001	
*P* for middle income^e^		0.94			0.21			0.91				
*P* for highest income^e^		0.25			0.80			0.90				
Meat (g/1,000 kcal)												
Lowest income	669	38.9	1.5	617	44.9	1.7	451	50.6	2.4	0.026	<0.001	
Middle income	1,824	41.6	1.1	1,735	45.9	1.4	1,340	53.5	1.5	0.009	<0.001	0.59
Highest income	807	42.5	1.5	814	49.2	1.7	878	53.9	1.8	<0.001	<0.001	
*P* for middle income^e^		0.096			0.61			0.25				
*P* for highest income^e^		0.072			0.059			0.27				
Egg (g/1,000 kcal)												
Lowest income	669	22.1	0.9	617	18.5	1.0	451	23.4	1.4	0.025	0.043	
Middle income	1,824	20.5	0.7	1,735	20.3	0.6	1,340	23.5	0.9	0.27	<0.001	0.29
Highest income	807	19.0	0.9	814	20.3	1.0	878	22.0	1.1	0.77	0.004	
*P* for middle income^e^		0.18			0.091			0.92				
*P* for highest income^e^		0.017			0.21			0.47				
Soy and other beans (g/1,000 kcal)												
Lowest income	669	32.5	2.2	617	34.1	2.1	451	36.0	3.0	0.94	0.43	
Middle income	1,824	34.2	1.6	1,735	35.9	1.7	1,340	35.2	1.9	0.15	0.16	0.83
Highest income	807	34.9	2.0	814	36.0	2.1	878	38.3	2.4	0.31	0.067	
*P* for middle income^e^		0.47			0.45			0.82				
*P* for highest income^e^		0.40			0.52			0.56				
Milk and dairy products (g/1,000 kcal)												
Lowest income	669	59.0	3.3	617	57.2	4.3	451	60.4	4.3	0.48	0.15	
Middle income	1,824	63.7	2.4	1,735	65.1	2.6	1,340	67.5	2.7	0.53	0.15	0.90
Highest income	807	67.7	3.4	814	72.7	3.5	878	72.3	3.5	0.29	0.55	
*P* for middle income^e^		0.23			0.074			0.12				
*P* for highest income^e^		0.061			0.002			0.029				
Snacks and confectionaries (g/1,000 kcal)												
Lowest income	453	13.5	1.2	423	14.2	1.2	281	15.6	1.4	0.31	0.045	
Middle income	1,715	15.1	0.9	1,620	16.5	1.0	1,259	16.4	1.0	0.34	0.40	0.37
Highest income	754	16.7	1.2	758	17.3	1.4	828	16.8	1.3	0.80	0.34	
*P* for middle income^e^		0.21			0.08			0.60				
*P* for highest income^e^		0.053			0.07			0.52				
Alcoholic beverages (g/1,000 kcal)												
Lowest income	453	23.7	3.3	423	30.4	4.1	281	37.3	5.7	0.095	0.082	
Middle income	1,715	25.9	2.8	1,620	32.7	3.4	1,259	35.1	3.9	0.0031	0.047	0.92
Highest income	754	31.6	4.1	758	35.3	4.9	828	41.1	5.0	0.13	0.14	
*P* for middle income^e^		0.52			0.63			0.70				
*P* for highest income^e^		0.083			0.43			0.59				
Tea, coffee, and other soft drinks (g/1,000 kcal)												
Lowest income	453	357.1	13.8	423	301.1	14.4	281	364.2	16.7	0.0022	0.98	
Middle income	1,715	365.6	10.7	1,620	352.0	11.1	1,259	345.0	11.0	0.066	0.16	0.10
Highest income	754	362.3	13.7	758	344.3	13.2	828	338.6	12.6	0.063	0.38	
*P* for middle income^e^		0.54			<0.001			0.27				
*P* for highest income^e^		0.77			0.014			0.23				

SE, standard error.

^a^Estimated by SAS PROC SURVEYREG with STRATA (for prefecture) and CLUSTER (for unit blocks) statements. The following confounding variables were adjusted: age (20–29, 30–39, 40–49, 50–59, 60–69, and ≥70 years), occupation (professional/manager, sales/service/clerical, security/transportation/labor, and non-worker), and number of participants from the same household (1, 2, ≥3).

^b^Participants were categorized into three groups according to income: lowest (<2 million yen), middle (2 to <6 million yen), and highest (≥6 million yen).

^c^Survey year as dummy variables using 2010 survey as reference were included in the model with adjustment for above mentioned variables.

^d^*P*-values for *the income (dummy variables)* × *survey year (dummy variables)* interaction terms were calculated with adjustment for the above-mentioned variables; a significant interaction meant that the *income-related differences* in food intake changed over time.

^e^Income as dummy variables using the lowest group as a reference were included in the model with adjustment for above mentioned variables.

For other food groups, the differences in intake among income groups did not change significantly over time. Meat intake was higher in the 2014 and 2018 surveys regardless of sex and income. In the middle- and the highest-income groups, the intakes of fruits as well as fish and shellfish were lower in the 2018 survey. These results were not changed after liming participants to one man and one woman from the same household (eTable 2 and eTable 3).

## DISCUSSION

To the best of our knowledge, this is the first study to evaluate the time trends of income-related differences in food intake in Japanese adults. Despite several economic changes affected by the 2008 Great Recession, the Great East Japan earthquake 2011, and consumption tax increase in 2014, widening of income-related differences in food intakes was not observed during survey period. Lower cereal intake in the middle and highest-income groups was stable over time. Unexpectedly, in 2018, the vegetable intake decreased among women in the middle- and highest-income groups, and the tendency of a higher vegetable intake in women with the highest income disappeared over time. Egg intake was lower in men with the middle and the highest income as per the 2010 survey, but a higher egg intake was observed in men with the middle income than in those with the lowest income in 2018. The tendency for higher intake of milk and dairy products in men with the highest income was stable over time.

Our study findings that indicate a higher cereal consumption in the low-income group were supported by studies conducted in Japan,^9^^,^^45^ China,^6^ and Korea,^46^ although non-significant or opposite relationships have been reported in studies conducted in other countries (eg, the United States, Australia, and Canada).^7^^,^^47^^,^^48^ Cereals, particularly refined cereals, are known for their low cost and low nutrient density at the same energy.^49^ Japanese people with a low income have limited food budgets and mainly consume cereal and a low variety of foods.^41^ Therefore, increasing the cereal intake is an easy way to increase satiety without increasing dietary costs in Japan, and it might result in a low-nutrient-density diet in low-income individuals. Public support is needed to help low-income individuals consume cereals with nutrient-rich foods.

In contrast, the tendency for higher vegetable consumption in women with the highest income disappeared over time. Among women with the middle and the highest income, vegetable intake decreased in 2018. Therefore, income-related differences in vegetable intake might tend to be narrowed, not due to the increased intake among women with a low income, but rather due to the decreased intake among women with a high income. In addition, in 2018, the fruit intake decreased in the higher-income groups. Considering the inverse relationship between fruit and vegetable intake and the risk of cardiovascular disease, cancer, and mortality,^50^ these dietary changes may have an unfavorable impact on the health of higher-income groups.

Previous studies revealed inconsistent results for the time trends of income-related differences in the consumption of fruits^21^^,^^24^ and vegetables^51^; this inconsistency might partly be explained by the stage of economic development and degree of urbanization. In Japan, working time is slightly increasing in the women with higher household income.^52^ For example, mean working minutes per day on weekdays was 219 in 2011, 234 in 2016, and 259 in 2021, among married women with household income of 7–9.99 million yen.^52^ Considering the possible association between longer working hours and lower vegetable intake in women,^53^ changes in working hours during the study period may partly explain the decline in vegetable intake in women with a higher income. These results suggest that social support is needed to remove the barriers concerning the consumption of fruits and vegetables regardless of income level. Moreover, changes in dietary habits should be monitored even in the high-income women whose diet quality is suggested to be the highest in the Japanese population.^10^

Although, men in the middle- and highest-income groups had low egg intake compared to those with the lowest income in 2010, the opposite tendency of egg intake according to income levels was observed in 2018. Egg intake increased in men in the middle- and high-income groups during the survey period. Although we do not know the reason of such findings, one assumption would be the sales price changes over these years. The average price of one pack of eggs (usually ten eggs per pack) in major cities in Japan increased slightly during the study period, comprising 202 yen in 2010, 227 yen in 2014, and 221 yen in 2018.^54^ This may explain the reason that low-income group did not increase egg consumption given they are more price-sensitive. Since the income-related difference in egg intake was not supported by previous studies,^6^^,^^46^^,^^55^ future studies are needed to confirm our study findings. Moreover, considering that the difference in egg intake among the income categories was around 3 g/1,000 kcal in this study, the probable impact of income-related differences in egg intake might have a small impact on health outcomes in the Japanese population. The positive relationship between income and milk and dairy products is supported by previous study findings.^6^^,^^11^^,^^46^^,^^48^^,^^56^ Japanese dairy consumers are more likely to have adequate calcium intake and inadequate saturated fats consumption.^57^ Further studies using detailed food grouping methods are needed as the relationship between income and low-fat milk intake differs from that of full-fat milk intake.^47^

This study had several limitations. First, only 65–70% of the sampled households took part in the survey. During the observational period, the household-level participation rate and proportion of low-income individuals slightly decreased. If low-income individuals with higher or lower dietary intake were less likely to participate in the survey or provide income information, the relationship between the income and food intake might be biased. Second, in the NHNS, information on household income is obtained using only three or four choices. Therefore, equivalized household income cannot be calculated to reflect the difference in household size. Moreover, the scientific basis of the cutoff-values of the income (<2 million yen, 2 to <6 million yen, ≥6 million yen) used in the question is not explained in the NHNS. Additionally, a precise definition of “household income,” such as whether to include income sources other than salary income, was not provided in the questionnaire. Collecting information on household income with more detailed choices and clear definitions in the NHNS is considered useful for planning population and high-risk approach interventions in Japan. Third, self-reported dietary assessments are subject to systematic and random errors. Food intake estimated from a 1-day dietary record does not likely represent the habitual food intake of the study participants. More importantly, the ability of household-based dietary records to estimate the energy and nutrient intake has been validated among female university students alone^39^ and not in women of other age groups or men. In addition, this study failed to assess the validity of the estimated food intake.^39^ Furthermore, previous studies have reported that under-reporting of energy intake was more prevalent in individuals with lower income than in those with higher income.^58^^,^^59^ Although food intake was adjusted for energy intake, the difference in the reporting accuracy of dietary intake between income levels may have affected our findings. Fourth, the STFC used for food coding was different among the three surveys, which might have affected the estimation of food groups and energy intake. However, the food grouping methods and cooking status of the calculated food intake did not dramatically change after the 2001 survey. Although no study has compared energy intakes estimated by the 5th and 2010 editions, it was suggested that the percentage difference in energy intake estimated by the 2010 and 2015 editions was small (1%).^60^ Fifth, although education is strong determinant of future employment and income,^61^ the models were not adjusted for education since the information has not been collected in the NHNS. Considering that education might have stronger associations with food intakes than income^62^ further studies are needed to determine how to monitor socioeconomic differences in dietary habits in Japanese. Sixth, given economic crisis, as well as related unemployment, could negatively impact dietary intake especially in individuals with low-socioeconomic status,^15^ a longer study period before the 2008 Great Recession might clarify the income-related differences in dietary habits of the Japanese population. Future studies using longer period data are needed.

In conclusion, despite economic challenges that could affect the most vulnerable people, widening of income-related differences in food intakes was not observed during the survey period. The tendency for lower cereal intake in the higher-income groups was stable over time in both sexes. Unexpectedly, vegetable intake decreased in women in the middle- and high-income groups in 2018, and the tendency of higher vegetable intake in women with the highest income disappeared over time. Further studies using data over a longer period with a detailed questionnaire on income are needed to determine change of the relationship between income and food intake over time.

## SUPPLEMENTARY MATERIAL

eTable 1. Characteristics of individuals included in the analyses and those who excluded from the analyses due to missing information of income: the 2010, 2014, and 2018 National Health and Nutrition Survey, Japan

eTable 2. Adjusted mean intakes^a^ of food groups according to income^b^ and survey year (men)

eTable 3. Adjusted mean intakes^a^ of food groups according to income^b^ and survey year (women)

## References

[1] Mackenbach JP, Stirbu I, Roskam AJ, . Socioeconomic inequalities in health in 22 European countries. N Engl J Med. 2008;358:2468–2481. 10.1056/NEJMsa0707519 18525043

[2] Jarvandi S, Yan Y, Schootman M. Income disparity and risk of death: the importance of health behaviors and other mediating factors. PLoS One. 2012;7:e49929. 10.1371/journal.pone.0049929 23185488 PMC3501486

[3] Stringhini S, Dugravot A, Shipley M, . Health behaviours, socioeconomic status, and mortality: further analyses of the British Whitehall II and the French GAZEL prospective cohorts. PLoS Med. 2011;8:e1000419. 10.1371/journal.pmed.1000419 21364974 PMC3043001

[4] Nejatinamini S, Godley J, Minaker LM, . Quantifying the contribution of modifiable risk factors to socio-economic inequities in cancer morbidity and mortality: a nationally representative population-based cohort study. Int J Epidemiol. 2021;50:1498–1511. 10.1093/ije/dyab067 33846746

[5] Giskes K, Turrell G, Patterson C, Newman B. Socio-economic differences in fruit and vegetable consumption among Australian adolescents and adults. Public Health Nutr. 2002;5:663–669. 10.1079/PHN2002339 12372160

[6] Yang X, Hsu-Hage BH, Tian H, . The role of income and education in food consumption and nutrient intake in a Chinese population. Asia Pac J Clin Nutr. 1998;7:217–226. 24393675

[7] Hiza HAB, Casavale KO, Guenther PM, Davis CA. Diet quality of Americans differs by age, sex, race/ethnicity, income, and education level. J Acad Nutr Diet. 2013;113:297–306. 10.1016/j.jand.2012.08.011 23168270

[8] Maguire ER, Monsivais P. Socio-economic dietary inequalities in UK adults: an updated picture of key food groups and nutrients from national surveillance data. Br J Nutr. 2015;113:181–189. 10.1017/S0007114514002621 25399952 PMC4351901

[9] Nishi N, Horikawa C, Murayama N. Characteristics of food group intake by household income in the National Health and Nutrition Survey, Japan. Asia Pac J Clin Nutr. 2017;26:156–159. 10.6133/apjcn.102015.15 28049275

[10] Kurotani K, Ishikawa-Takata K, Takimoto H. Diet quality of Japanese adults with respect to age, sex, and income level in the National Health and Nutrition Survey, Japan. Public Health Nutr. 2020;23:821–832. 10.1017/S1368980019002088 31736456 PMC7282861

[11] Medina LPB, Barros MBA, Sousa NFDS, . Social inequalities in the food consumption profile of the Brazilian population: National Health Survey, 2013. Rev Bras Epidemiol. 2019;22(Suppl 02):E190011.SUPL.2. 10.1590/1980-549720190011.supl.2 31596382

[12] Rodrigues SSP, Caraher M, Trichopoulou A, de Almeida MDv. Portuguese households’ diet quality (adherence to Mediterranean food pattern and compliance with WHO population dietary goals): trends, regional disparities and socioeconomic determinants. Eur J Clin Nutr. 2008;62:1263–1272. 10.1038/sj.ejcn.1602852 17671445

[13] Padrão P, Laszczyńska O, Silva-Matos C, Damasceno A, Lunet N. Low fruit and vegetable consumption in Mozambique: results from a WHO STEPwise approach to chronic disease risk factor surveillance. Br J Nutr. 2012;107:428–435. 10.1017/S0007114511003023 21762541

[14] Popkin BM. Global nutrition dynamics: the world is shifting rapidly toward a diet linked with noncommunicable diseases. Am J Clin Nutr. 2006;84:289–298. 10.1093/ajcn/84.2.289 16895874

[15] Jenkins RH, Vamos EP, Taylor-Robinson D, Millett C, Laverty AA. Impacts of the 2008 Great Recession on dietary intake: a systematic review and meta-analysis. Int J Behav Nutr Phys Act. 2021;18:57. 10.1186/s12966-021-01125-8 33926455 PMC8084260

[16] Green R, Cornelsen L, Dangour AD, . The effect of rising food prices on food consumption: systematic review with meta-regression. BMJ. 2013;346:f3703. 10.1136/bmj.f3703 23775799 PMC3685509

[17] Sumar N, McLaren L. Impact on social inequalities of population strategies of prevention for folate intake in women of childbearing age. Am J Public Health. 2011;101:1218–1224. 10.2105/AJPH.2010.300018 21566037 PMC3110217

[18] McGill R, Anwar E, Orton L, . Are interventions to promote healthy eating equally effective for all? Systematic review of socioeconomic inequalities in impact. BMC Public Health. 2015;15:457. 10.1186/s12889-015-1781-7 25934496 PMC4423493

[19] Olstad DL, Teychenne M, Minaker LM, . Can policy ameliorate socioeconomic inequities in obesity and obesity-related behaviours? A systematic review of the impact of universal policies on adults and children. Obes Rev. 2016;17:1198–1217. 10.1111/obr.12457 27484468

[20] Frohlich KL, Potvin L. Transcending the known in public health practice: the inequality paradox: the population approach and vulnerable populations. Am J Public Health. 2008;98:216–221. 10.2105/AJPH.2007.114777 18172133 PMC2376882

[21] Rehm CD, Peñalvo JL, Afshin A, Mozaffarian D. Dietary Intake Among US Adults, 1999–2012. JAMA. 2016;315:2542–2553. 10.1001/jama.2016.7491 27327801 PMC6287627

[22] Wang Z, Gordon-Larsen P, Siega-Riz AM, . Sociodemographic disparity in the diet quality transition among Chinese adults from 1991 to 2011. Eur J Clin Nutr. 2017;71:486–493. 10.1038/ejcn.2016.179 27677363 PMC5373942

[23] Mello AV, Sarti FM, Pereira JL, . Determinants of inequalities in the quality of Brazilian diet: trends in 12-year population-based study (2003–2015). Int J Equity Health. 2018;17:72. 10.1186/s12939-018-0784-2 29879999 PMC5992855

[24] Olstad DL, Leech RM, Livingstone KM, . Are dietary inequalities among Australian adults changing? a nationally representative analysis of dietary change according to socioeconomic position between 1995 and 2011–13. Int J Behav Nutr Phys Act. 2018;15:30. 10.1186/s12966-018-0666-4 29606145 PMC5879763

[25] Scholes S, Bajekal M, Love H, . Persistent socioeconomic inequalities in cardiovascular risk factors in England over 1994–2008: a time-trend analysis of repeated cross-sectional data. BMC Public Health. 2012;12:129. 10.1186/1471-2458-12-129 22333887 PMC3342910

[26] Murayama N, Ishida H, Yamamoto T, . Household income is associated with food and nutrient intake in Japanese schoolchildren, especially on days without school lunch. Public Health Nutr. 2017;20:2946–2958. 10.1017/S1368980017001100 28851478 PMC10261441

[27] Statistics Bureau. Ministry of internal affairs and communications. Labour Force Survey. https://www.stat.go.jp/english/data/roudou/report/index.html.

[28] Katanoda K, Matsumura Y. National Nutrition Survey in Japan—its methodological transition and current findings. J Nutr Sci Vitaminol (Tokyo). 2002;48:423–432. 10.3177/jnsv.48.423 12656220

[29] Saito A, Imai S, Htun NC, . The trends in total energy, macronutrients and sodium intake among Japanese: findings from the 1995–2016 National Health and Nutrition Survey. Br J Nutr. 2018;120:424–434. 10.1017/S0007114518001162 29860946

[30] Office of Nutrition Cancer Measures and Health Promotion. Division Health service Bureau. Ministry of Health Labour and Welfare. The National Health and Nutrition Survey in Japan, 2010. https://www.mhlw.go.jp/bunya/kenkou/eiyou/dl/h22-houkoku-01.pdf. 2010.

[31] Office of Nutrition Cancer Measures and Health Promotion. Division Health service Bureau. Ministry of Health Labour and Welfare. The National Health and Nutrition Survey in Japan, 2014. https://www.mhlw.go.jp/bunya/kenkou/eiyou/dl/h26-houkoku.pdf. 2014.

[32] National Institutes of Biomedical Innovation Health and Nutrition. The National Health and Nutrition Survey (NHNS) Japan, 2014 Summary. https://www.nibiohn.go.jp/eiken/kenkounippon21/download_files/eiyouchousa/2014.pdf. 2014.

[33] Office of Nutrition Cancer Measures and Health Promotion. Division Health service Bureau. Ministry of Health Labour and Welfare. The National Health and Nutrition Survey in Japan, 2018. https://www.mhlw.go.jp/content/000681200.pdf. 2018.

[34] National Institutes of Biomedical Innovation Health and Nutrition. The National Health and Nutrition Survey (NHNS) Japan, 2018 Summary. https://www.nibiohn.go.jp/eiken/kenkounippon21/download_files/eiyouchousa/2018.pdf. 2018.

[35] Science and Technology Agency. *Standard Tables of Food Composition in Japan (Fifth Revised and Enlarged Edition)*. The Printing Bureau, Ministry of Finance; 2005.

[36] The Council for Science and Technology Ministry of Education Culture Sports Science and Technology. *Standard Tables of Food Composition in Japan 2010*. Official Gazette Co-operation; 2010.

[37] The Council for Science and Technology Ministry of Education Culture Sports Science and Technology. *Standard Tables of Food Composition in Japan 2015 (7th revised edition)*. National Printing Bureau; 2015.

[38] Walter Willett. *Nutritional epidemiology 3rd ed.* Oxford University Press; 2013.

[39] Iwaoka F, Yoshiike N, Date C, Shimada T, Tanaka H. A validation study on a method to estimate nutrient intake by family members through a household-based food-weighing survey. J Nutr Sci Vitaminol (Tokyo). 2001;47:222–227. 10.3177/jnsv.47.222 11575577

[40] Ministry of Health Labour and Welfare. Basic Survey on Wage Structure. https://www.mhlw.go.jp/toukei/list/chinginkouzou_b.html#10. 2020.

[41] Okubo H, Murakami K, Sasaki S. Monetary value of self-reported diets and associations with sociodemographic characteristics and dietary intake among Japanese adults: analysis of nationally representative surveys. Public Health Nutr. 2016;19:3306–3318. 10.1017/S1368980016001695 27357725 PMC10270988

[42] Tsubota-Utsugi M, Kikuya M, Satoh M, . Living situations associated with poor dietary intake among healthy Japanese elderly: the Ohasama Study. J Nutr Health Aging. 2015;19:375–382. 10.1007/s12603-015-0456-525809800

[43] Tanaka R, Tsuji M, Asakura K, . Variation in men’s dietary intake between occupations, based on data from the Japan Environment and Children’s Study. Am J Men Health. 2018;12:1621–1634. 10.1177/1557988318780847 29890875 PMC6142127

[44] Kobayashi S, Asakura K, Suga H, Sasaki S. Cohabitational effect of grandparents on dietary intake among young Japanese women and their mothers living together. A multicenter cross-sectional study. Appetite. 2015;91:287–297. 10.1016/j.appet.2015.04.059 25916625

[45] Nagahata T, Nakamura M, Ojima T, . Relationships among food group intakes, household expenditure, and education attainment in a general Japanese population: NIPPON DATA2010. J Epidemiol. 2018;28(Suppl 3):S23–S28. 10.2188/jea.JE20170248 29503382 PMC5825688

[46] Hur I, Jang MJ, Oh K. Food and nutrient intakes according to income in Korean men and women. Osong Public Health Res Perspect. 2011;2:192–197. 10.1016/j.phrp.2011.11.044 24159472 PMC3767089

[47] Smith AM, Baghurst KI. Public health implications of dietary differences between social status and occupational category groups. J Epidemiol Community Health. 1992;46:409–416. 10.1136/jech.46.4.409 1431718 PMC1059611

[48] Ricciuto LE, Tarasuk VS. An examination of income-related disparities in the nutritional quality of food selections among Canadian households from 1986–2001. Soc Sci Med. 2007;64:186–198. 10.1016/j.socscimed.2006.08.020 17030372

[49] Maillot M, Darmon N, Darmon M, Lafay L, Drewnowski A. Nutrient-dense food groups have high energy costs: an econometric approach to nutrient profiling. J Nutr. 2007;137:1815–1820. 10.1093/jn/137.7.1815 17585036

[50] Yip CSC, Chan W, Fielding R. The associations of fruit and vegetable intakes with burden of diseases: a systematic review of meta-analyses. J Acad Nutr Diet. 2019;119:464–481. 10.1016/j.jand.2018.11.007 30639206

[51] Roos E, Talala K, Laaksonen M, . Trends of socioeconomic differences in daily vegetable consumption, 1979–2002. Eur J Clin Nutr. 2008;62:823–833. 10.1038/sj.ejcn.1602798 17522606

[52] Statistics Bureau of Japan. Ministry of Internal Affairs and Communications. Survey on time use and leisure activities. https://www.stat.go.jp/english/data/shakai/index.html.

[53] Oono F, Matsuura N, Saito A, . Association of hours of paid work with dietary intake and quality in Japanese married women: a cross-sectional study. Nutrients. 2021;13:3005. 10.3390/nu13093005 34578884 PMC8466932

[54] Price Statistics Office Statistics Bureau. Retail Price Survey (in Japanese). https://www.stat.go.jp/data/kouri/index.html.

[55] Conrad Z, Johnson LK, Roemmich JN, Juan W, Jahns L. Time trends and patterns of reported egg consumption in the U.S. by sociodemographic characteristics. Nutrients. 2017;9:333. 10.3390/nu9040333 28350345 PMC5409672

[56] Tarasuk V, Fitzpatrick S, Ward H. Nutrition inequities in Canada. Appl Physiol Nutr Metab. 2010;35:172–179. 10.1139/H10-002 20383227

[57] Saito A, Okada E, Tarui I, Matsumoto M, Takimoto H. The Association between milk and dairy products consumption and nutrient intake adequacy among Japanese adults: analysis of the 2016 National Health and Nutrition Survey. Nutrients. 2019;11:2361. 10.3390/nu11102361 31623382 PMC6835801

[58] Murakami K, Livingstone MBE. Prevalence and characteristics of misreporting of energy intake in US adults: NHANES 2003–2012. Br J Nutr. 2015;114:1294–1303. 10.1017/S0007114515002706 26299892

[59] Kye S, Kwon SO, Lee SY, . Under-reporting of energy intake from 24-hour dietary recalls in the Korean National Health and Nutrition Examination Survey. Osong Public Health Res Perspect. 2014;5:85–91. 10.1016/j.phrp.2014.02.002 24955317 PMC4064631

[60] Saito A, Okada E, Matsumoto M, Takimoto H. Impact of updated standard tables of food composition on nutrient intakes in Japan. J Food Compos Anal. 2019;79:5–11. 10.1016/j.jfca.2019.02.008

[61] Galobardes B, Shaw M, Lawlor DA, Lynch JW, Davey Smith G. Indicators of socioeconomic position (part 1). J Epidemiol Community Health. 2006;60:7–12. 10.1136/jech.2004.023531 16361448 PMC2465546

[62] Murakami K, Miyake Y, Sasaki S, . Education, but not occupation or household income, is positively related to favorable dietary intake patterns in pregnant Japanese women: the Osaka Maternal and Child Health Study. Nutr Res. 2009;29:164–172. 10.1016/j.nutres.2009.02.002 19358930

