# Supplementary material for: Time Trends in Income-related Differences in Food Group Intakes: The National Health and Nutrition Survey, Japan in 2010, 2014, and 2018
Source: J Epidemiol. 2024 Feb 5;34(2):76–86. doi: 10.2188/jea.JE20220220 (PMC10751188; doi:10.2188/jea.JE20220220)
Supplement: Supplementary file 1 [file je-34-076-s001.pdf]

eTable 1. Characteristics of individuals included in the analyses and those who excluded from the analyses due to missing information of income: the 2010, 2014, and 2018 National Health and Nutrition Survey, Japan

	2010					2014					2018				
	Included in the analyses		Excluded from the analyses		*P*-value [a]	Included in the analyses		Excluded from the analyses		*P*-value [a]	Included in the analyses		Excluded from the analyses		*P*-value [a]
	n	%	n	%		n	%	n	%		n	%	n	%	
Sex	6,222	100.0	1793	100.0	0.32	5,967	100.0	1,771	100.0	0.52	5,037	100.0	1,605	100.0	0.23
Men	2,922	47.0	818	45.6		2,801	46.9	816	46.1		2,368	47.0	727	45.3	
Women	3,300	53.0	975	54.4		3,166	53.1	955	53.9		2,669	53.0	878	54.7	
Age	6,222	100.0	1793	100.0	0.001	5,967	100.0	1,771	100.0	<0.001	5,037	100.0	1,605	100.0	<0.001
20–29 years	539	8.7	212	11.8		396	6.6	196	11.1		367	7.3	169	10.5	
30–39 years	922	14.8	282	15.7		676	11.3	272	15.4		522	10.4	258	16.1	
40–49 years	931	15.0	270	15.1		903	15.1	273	15.4		831	16.5	249	15.5	
50–59 years	1,020	16.4	279	15.6		916	15.4	260	14.7		825	16.4	219	13.6	
60–69 years	1,363	21.9	358	20.0		1,431	24.0	329	18.6		1,047	20.8	277	17.3	
≥70 years	1,447	23.3	392	21.9		1,645	27.6	441	24.9		1,445	28.7	433	27.0	
Household income	6,222	100.0	692	100.0	0.015	5,967	100.0	954	100.0	0.41	5,037	100.0	852	100.0	0.035
<2 million yen	1,122	18.0	111	16.0		1,040	17.4	183	19.2		732	14.5	150	17.6	
2 to <6 million yen	3,539	56.9	433	62.6		3,355	56.2	528	55.3		2,599	51.6	440	51.6	
≥6 million yen	1,561	25.1	148	21.4		1,572	26.3	243	25.5		1,706	33.9	262	30.8	
Occupation	6,222	100.0	1339	100.0	<0.001	5,967	100.0	1,249	100.0	0.69	5,037	100.0	1,094	100.0	0.045
Professional/manager	963	15.5	176	13.1		857	14.4	170	13.6		901	17.9	165	15.1	
Sales/service/clerical	1,597	25.7	298	22.3		1,443	24.2	292	23.4		1,323	26.3	270	24.7	
Security/transportation/labor	1,099	17.7	277	20.7		1,065	17.8	237	19.0		823	16.3	196	17.9	
Non-worker	2,563	41.2	588	43.9		2,602	43.6	550	44.0		1,990	39.5	463	42.3	
Number of participants per household	6,222	100.0	1793	100.0	0.14	5,967	100.0	1,771	100.0	0.067	5,037	100.0	1,605	100.0	0.001
1	599	9.6	196	10.9		657	11.0	230	13.0		630	12.5	226	14.1	
2	1,965	31.6	533	29.7		1,954	32.7	559	31.6		1,653	32.8	585	36.4	
≥3	3,658	58.8	1064	59.3		3,356	56.2	982	55.4		2,754	54.7	794	49.5	
Current smoking [b]	6,110	100.0	1690	100.0	0.002	5,914	100.0	1,707	100.0	0.018	4,994	100.0	1,554	100.0	0.07
No	4,880	79.9	1290	76.3		4,791	81.0	1,339	78.4		4,128	82.7	1,253	80.6	
Yes	1,230	20.1	400	23.7		1,123	19.0	368	21.6		866	17.3	301	19.4	
Harmful drinking habits [c]	6,162	100.0	1711	100.0	0.38	5,919	100.0	1,709	100.0	0.26	4,995	100.0	1,554	100.0	0.23
No	5,464	88.7	1530	89.4		5,191	87.7	1,516	88.7		4,399	88.1	1,386	89.2	
Yes	698	11.3	181	10.6		728	12.3	193	11.3		596	11.9	168	10.8	
Food intake, n	6,222		1007			5,967		760			5,037		706		
	Median	IQR	Median	IQR		Median	IQR	Median	IQR		Median	IQR	Median	IQR	
Cereals (g/1,000 kcal)	238.0	(190.3–289.3)	252.4	(200.0–304.5)	<0.001	232.0	(182.6–285.7)	252.9	(199.6–308.4)	<0.001	215.3	(167.1–267.1)	228.0	(178.9–275.9)	<0.001
Vegetables (g/1,000 kcal)	151.3	(98.2–216.2)	156.6	(103.4–225.5)	0.027	156.3	(102.3–221.4)	137.9	(92.4–199)	<0.001	143.2	(91.6–206.5)	136.6	(90.3–200.8)	0.19
Fruits (g/1,000 kcal)	32.9	(0.0–84.5)	18.0	(0.0–71.9)	<0.001	33.8	(0.0–88.3)	26.3	(0.0–77.5)	0.023	29.7	(0.0–79.4)	12.4	(0.0–77.0)	0.032
Fishes and shellfishes (g/1,000 kcal)	36.3	(9.4–64.5)	37.2	(9.5–63.4)	0.93	34.4	(7.6–61.4)	31.4	(6.8–59.2)	0.26	30.6	(4.4–57.6)	31.9	(3.0–57.4)	0.89

Meats (g/1,000 kcal)	36.8	(16.1–60.8)	35.8	(15.4–62.8)	0.84	40.3	(17.9–66.3)	40.5	(15.8–64.6)	0.39	47.5	(23.8–75.5)	46.9	(23.7–73.7)	0.99
Eggs (g/1,000 kcal)	17.2	(1.0–30.5)	14.4	(0.0–29.4)	0.005	16.9	(0.6–28.9)	15.7	(0.0–30.3)	0.81	21.3	(2.0–34.0)	20.1	(0.0–33.4)	0.12
Pulses (g/1,000 kcal)	20.2	(0.0–47.4)	18.3	(0.0–48.5)	0.43	22.1	(0.0–50.4)	16.2	(0.0–43.4)	<0.001	22.9	(1.4–51.0)	22.0	(1.9–47.9)	0.81
Milk and dairy products (g/1,000 kcal)	15.6	(0.0–88.0)	5.1	(0.0–77.3)	<0.001	26.6	(0.0–94.6)	10.9	(0.0–86.6)	0.003	34.5	(0.0–94.7)	25.7	(0.0–89.9)	0.003

[a] Chi-square test for categorical variables and Wilcoxon rank sum test for continuous variables.

[b] Participants who responded “smoking every day” or “occasionally smoking”.

[c] Defined according to the definition used in the NHNS report: : participants who responded question about drinking frequencies and amount as “every day and ≥2 units/day”, “5–6 days/week and ≥2 units/day”, “3–4 days/week and ≥3 units/day”, “1–2 days/week and ≥5 units/day”, or “1–3 days/month and ≥5 units/day” for men, and “every day and ≥1 unit/day”, “5–6 days/week and ≥1 unit/day”, “3–4 days/week and ≥1 units/day”, “1–2 days/week and ≥3 units/day”, or “1–3 days/month and ≥5 units/day” for women.

eTable 2. Adjusted mean intakes [a] of food groups according to income [b] and survey year (men)

	2010（n=2,403）			2014（n=2,315）			2018（n=2,042）			*P* for 2014 [c]	*P* for 2018 [c]	*P*-interaction [d]
	n	Adjusted mean	SE	n	Adjusted mean	SE	n	Adjusted mean	SE			
Cereals (g/1,000 kcal)												
Lowest income	398	269.0	4.5	379	268.2	5.3	260	244.4	5.6	0.60	<0.001	
Middle income	1,419	250.8	2.4	1,348	243.0	3.3	1,115	229.9	3.0	0.02	<0.001	0.42
Highest income	586	243.5	4.1	588	236.5	4.7	667	229.9	3.6	0.32	<0.001	
p for middle income [e]		<0.001			<0.001			0.03				
p for highest income [e]		<0.001			<0.001			0.035				
Vegetables (g/1,000 kcal)												
Lowest income	398	135.2	4.9	379	125.4	5.4	260	130.0	5.7	0.19	0.25	
Middle income	1,419	134.6	2.6	1,348	138.4	3.3	1,115	131.4	2.6	0.76	0.013	0.14
Highest income	586	145.9	3.9	588	155.6	4.6	667	133.1	3.7	0.12	0.0089	
p for middle income [e]		0.91			0.03			0.81				
p for highest income [e]		0.078			<0.001			0.65				
Fruits (g/1,000 kcal)												
Lowest income	398	31.7	2.9	379	28.2	3.4	260	24.1	4.0	0.59	0.50	
Middle income	1,419	37.7	1.6	1,348	36.9	2.0	1,115	31.5	1.7	0.92	0.024	0.42
Highest income	586	42.3	2.4	588	37.5	2.6	667	31.2	1.9	0.22	<0.001	
p for middle income [e]		0.043			0.030			0.10				
p for highest income [e]		0.0062			0.04			0.15				
Fishes and shellfishes (g/1,000 kcal)												
Lowest income	398	39.3	1.9	379	38.1	2.3	260	33.9	2.6	0.44	0.10	
Middle income	1,419	39.2	1.1	1,348	37.9	1.3	1,115	32.0	1.2	0.16	<0.001	0.30
Highest income	586	41.7	1.9	588	35.0	2.0	667	31.6	1.8	0.0018	<0.001	
p for middle income [e]		0.95			0.95			0.52				
p for highest income [e]		0.39			0.30			0.48				
Meats (g/1,000 kcal)												
Lowest income	398	41.5	1.7	379	51.5	2.2	260	57.8	2.9	0.0016	<0.001	
Middle income	1,419	45.5	1.1	1,348	52.2	1.4	1,115	59.7	1.5	<0.001	<0.001	0.48
Highest income	586	46.3	1.7	588	55.6	1.7	667	59.7	2.0	<0.001	<0.001	
p for middle income [e]		0.015			0.76			0.58				
p for highest income [e]		0.043			0.14			0.60				
Eggs (g/1,000 kcal)												
Lowest income	398	21.2	1.0	379	15.3	1.0	260	17.0	1.3	<0.001	0.082	
Middle income	1,419	17.3	0.5	1,348	17.2	0.6	1,115	21.2	0.8	0.45	<0.001	<0.001

Highest income	586	16.7	0.8	588	18.0	0.8	667	20.5	1.1	0.20	0.0047	
p for middle income [e]		<0.001			0.077			0.0052				
p for highest income [e]		<0.001			0.057			0.045				
Soy and other beans (g/1,000 kcal)												
Lowest income	398	30.4	2.4	379	31.1	2.8	260	28.1	3.4	0.65	0.80	
Middle income	1,419	28.4	1.3	1,348	29.4	1.5	1,115	29.8	1.5	0.54	0.35	0.92
Highest income	586	27.5	1.9	588	31.5	1.8	667	31.9	2.2	0.14	0.15	
p for middle income [e]		0.94			0.57			0.63				
p for highest income [e]		0.15			0.90			0.37				
Milk and dairy products (g/1,000 kcal)												
Lowest income	398	34.0	3.4	379	38.8	4.3	260	42.3	4.8	0.17	0.025	
Middle income	1419	42.7	2.4	1,348	46.3	2.3	1,115	48.6	2.4	0.040	0.0072	0.79
Highest income	586	48.5	3.7	588	47.1	2.9	667	54.5	3.4	0.70	0.31	
p for middle income [e]		0.0091			0.085			0.24				
p for highest income [e]		0.0019			0.089			0.033				
Snacks and confectionaries (g/1,000 kcal)												
Lowest income	398	10.3	1.1	379	10.1	1.2	260	8.9	1.3	0.99	0.97	
Middle income	1,419	9.0	0.6	1,348	10.2	0.9	1,115	9.8	0.7	0.047	0.077	0.63
Highest income	586	9.0	1.0	588	9.3	1.0	667	10.5	1.0	0.80	0.29	
p for middle income [e]		0.25			0.93			0.53				
p for highest income [e]		0.36			0.61			0.34				
Alcoholic beverages (g/1,000 kcal)												
Lowest income	398	73.8	7.8	379	70.1	9.0	260	100.4	9.7	0.92	0.13	
Middle income	1,419	90.0	5.0	1,348	101.7	6.1	1,115	88.0	5.4	0.033	0.64	0.14
Highest income	586	101.4	8.4	588	106.2	8.6	667	97.9	8.3	0.65	0.83	
p for middle income [e]		0.044			0.0015			0.24				
p for highest income [e]		0.0096			0.0048			0.83				
Non-alcoholic beverages (g/1,000 kcal)												
Lowest income	398	263.7	13.0	379	253.6	16.3	260	279.4	15.4	0.28	0.69	
Middle income	1,419	281.5	7.7	1,348	280.8	9.6	1,115	266.5	8.4	0.14	0.32	0.60
Highest income	586	276.2	11.5	588	294.7	17.7	667	272.9	10.6	0.83	0.84	
p for middle income [e]		0.17			0.073			0.43				
p for highest income [e]		0.46			0.043			0.73				

SE, standard error.

[a] Analyses were conducted limiting participants to a single male from the same household. Estimated by SAS PROC SURVEYREG with STRATA (for prefecture) and CLUSTER (for unit blocks) statements. The following variables were adjusted: age (20–29, 30–39, 40–49, 50–59, 60–69, and ≥70 years), occupation (professional/manager, sales/service/clerical, security/transportation/labor, and non-worker), and number of participants

from the same household (1, 2, or ≥3).

[b] Participants were categorized into three groups according to income: lowest (<2 million yen), middle (2 to <6 million yen), and highest (≥6 million yen).

[c] Survey year as dummy variables using 2010 as a reference were included in the model with adjustment for above mentioned variables.

[d] *P*-values for *the income (dummy variables)* × *survey year (dummy variables)* interaction terms were calculated with adjustment for the above-mentioned variables; a significant interaction meant that the *income-related differences* in food intake changed over time.

[e] Income as dummy variables using the lowest group as a reference were included in the model with adjustment for above mentioned variables.

eTable 3. Adjusted mean intakes [a] of food groups according to income [b] and survey year (women)

	2010（n=2,621）			2014（n=2,551）			2018（n=2,172）			*P* for 2014 [c]	*P* for 2018 [c]	*P*-interaction [d]
	n	Adjusted mean	SE	n	Adjusted mean	SE	n	Adjusted mean	SE			
Cereals (g/1,000 kcal)												
Lowest income	578	241.2	3.4	554	237.8	4.5	402	221.4	4.9	0.55	<0.001	
Middle income	1,460	226.6	2.6	1,394	217.8	3.0	1,117	209.7	3.4	0.11	<0.001	0.10
Highest income	583	217.4	3.6	603	207.6	3.7	653	201.4	4.4	0.13	<0.001	
p for middle income [e]		<0.001			<0.001			0.011				
p for middle income [e]		<0.001			<0.001			0.0016				
Vegetables (g/1,000 kcal)												
Lowest income	578	171.7	5.1	554	177.5	5.7	402	174.2	6.6	0.91	0.36	
Middle income	1,460	174.3	3.5	1,394	178.8	3.6	1,117	164.4	4.4	0.046	0.033	0.0083
Highest income	583	189.7	5.1	603	194.3	6.0	653	158.0	4.9	0.036	<0.001	
p for middle income [e]		0.64			0.84			0.12				
p for middle income [e]		0.010			0.033			0.039				
Fruits (g/1,000 kcal)												
Lowest income	578	54.4	3.4	554	51.6	3.6	402	47.5	3.9	0.77	0.47	
Middle income	1,460	60.8	2.5	1,394	59.0	2.4	1,117	50.6	2.3	0.77	0.003	0.90
Highest income	583	62.8	3.6	603	61.6	3.6	653	53.9	3.0	0.84	0.21	
p for middle income [e]		<0.001			0.062			0.48				
p for middle income [e]		<0.001			0.052			0.19				
Fishes and shellfishes (g/1,000 kcal)												
Lowest income	578	39.9	2.0	554	35.2	2.2	402	35.7	2.7	0.20	0.30	
Middle income	1,460	40.9	1.4	1,394	39.4	1.5	1,117	34.5	1.5	0.57	0.0023	0.097
Highest income	583	44.1	2.1	603	36.4	1.9	653	35.0	1.9	<0.001	<0.001	
p for middle income [e]		0.61			0.072			0.67				
p for middle income [e]		0.14			0.62			0.82				
Meats (g/1,000 kcal)												
Lowest income	578	39.3	1.5	554	46.6	2.0	402	50.8	2.3	0.0072	<0.001	
Middle income	1,460	42.5	1.2	1,394	46.2	1.4	1,117	53.4	1.5	0.022	<0.001	0.49
Highest income	583	43.1	1.6	603	49.2	1.8	653	53.2	1.8	0.0015	<0.001	
p for middle income [e]		0.041			0.86			0.26				
p for middle income [e]		0.061			0.29			0.39				
Eggs (g/1,000 kcal)												
Lowest income	578	21.3	1.0	554	17.8	1.1	402	23.2	1.4	0.060	0.026	
Middle income	1,460	19.6	0.7	1,394	19.3	0.7	1,117	23.1	0.9	0.37	<0.001	0.30

Highest income	583	18.9	0.8	603	19.9	1.1	653	21.5	1.0	0.96	0.020	
p for middle income [e]		0.15			0.14			0.99				
p for middle income [e]		0.074			0.14			0.35				
Soy and other beans (g/1,000 kcal)												
Lowest income	578	30.8	2.0	554	35.8	2.3	402	34.6	2.9	0.85	0.56	
Middle income	1,460	32.7	1.6	1,394	37.1	1.8	1,117	35.7	1.9	0.038	0.037	0.73
Highest income	583	34.9	2.0	603	35.2	2.1	653	38.8	2.6	0.57	0.061	
p for middle income [e]		0.37			0.62			0.75				
p for middle income [e]		0.11			0.86			0.30				
Milk and dairy products (g/1,000 kcal)												
Lowest income	578	56.5	3.5	554	56.4	4.4	402	58.4	4.6	0.47	0.15	
Middle income	1,460	61.9	2.6	1,394	65.5	2.7	1,117	67.2	3.0	0.33	0.067	0.83
Highest income	583	69.0	3.9	603	72.6	3.5	653	72.9	3.7	0.93	0.89	
p for middle income [e]		0.16			0.050			0.054				
p for middle income [e]		0.012			0.0018			0.011				
Snacks and confectionaries (g/1,000 kcal)												
Lowest income	578	13.8	1.2	554	14.0	1.2	402	14.9	1.6	0.42	0.11	
Middle income	1,460	15.8	1.0	1,394	16.6	1.1	1,117	16.4	1.0	0.88	0.84	0.56
Highest income	583	17.5	1.4	603	18.2	1.7	653	17.8	1.5	0.79	0.36	
p for middle income [e]		0.13			0.041			0.33				
p for middle income [e]		0.045			0.030			0.13				
Alcoholic beverages (g/1,000 kcal)												
Lowest income	578	25.6	4.0	554	30.1	4.7	402	38.1	6.4	0.27	0.14	
Middle income	1,460	26.5	3.2	1,394	34.0	3.9	1,117	35.7	4.2	0.0048	0.14	0.64
Highest income	583	29.0	4.4	603	36.2	5.7	653	38.7	5.6	0.040	0.30	
p for middle income [e]		0.82			0.45			0.70				
p for middle income [e]		0.55			0.40			0.94				
Non-alcoholic beverages (g/1,000 kcal)												
Lowest income	578	354.1	14.8	554	292.5	14.4	402	365.0	17.7	0.0021	0.74	
Middle income	1,460	364.7	11.4	1,394	344.5	12.2	1,117	349.2	10.9	0.048	0.049	0.090
Highest income	583	361.6	15.0	603	346.3	14.1	653	340.9	12.0	0.19	0.13	
p for middle income [e]		0.47			<0.001			0.38				
p for middle income [e]		0.69			0.0041			0.25				

SE, standard error.

[a] Analyses were conducted limiting participants to a single female from the same household. Estimated by SAS PROC SURVEYREG with STRATA (for prefecture) and CLUSTER (for unit blocks) statements. The following variables were adjusted: age (20–29, 30–39, 40–49, 50–59, 60–69, and ≥70 years), occupation (professional/manager, sales/service/clerical, security/transportation/labor, and non-worker), and number of participants from

the same household (one, two, or ≥ three).

[b] Participants were categorized into three groups according to income: lowest (<2 million yen), middle (2 to <6 million yen), and highest (≥6 million yen).

[c] Survey year as dummy variables using 2010 as a reference were included in the model with adjustment for above mentioned variables.

[d] *P*-values for *the income (dummy variables)* × *survey year (dummy variables)* interaction terms were calculated with adjustment for the above-mentioned variables; a significant interaction meant that the *income-related differences* in food intake changed over time.

[e] Income as dummy variables using the lowest group as a reference were included in the model with adjustment for above mentioned variables.
